# Organic Light-Emitting Diodes Based on Conjugation-Induced Thermally Activated Delayed Fluorescence Polymers: Interplay Between Intra- and Intermolecular Charge Transfer States

**DOI:** 10.3389/fchem.2019.00688

**Published:** 2019-10-23

**Authors:** Yungui Li, Qiang Wei, Liang Cao, Felix Fries, Matteo Cucchi, Zhongbin Wu, Reinhard Scholz, Simone Lenk, Brigitte Voit, Ziyi Ge, Sebastian Reineke

**Affiliations:** ^1^Dresden Integrated Center for Applied Physics and Photonic Materials (IAPP), Institute for Applied Physics, Technische Universität Dresden, Dresden, Germany; ^2^Ningbo Institute of Materials Technology & Engineering, Chinese Academy Sciences, Ningbo, China; ^3^Key Laboratory of Advanced Textile Materials and Manufacturing Technology, Ministry of Education, Zhejiang Sci-Tech University, Hangzhou, China; ^4^Leibniz-Institut für Polymerforschung Dresden e.V, Dresden, Germany; ^5^Organic Chemistry of Polymers, Technische Universität Dresden, Dresden, Germany

**Keywords:** thermally activated delayed fluorescence, electroluminescent polymer, exciplex, charge-transfer state, organic light-emitting diodes

## Abstract

In this work, interactions between different host materials and a blue TADF polymer named P1 are systematically investigated. In photoluminescence, the host can have substantial impact on the photoluminescence quantum yield (PLQY) and the intensity of delayed fluorescence (*Φ*_DF_), where more than three orders of magnitude difference of *Φ*_DF_ in various hosts is observed, resulting from a polarity effect of the host material and energy transfer. Additionally, an intermolecular charge-transfer (CT) emission with pronounced TADF characteristics is observed between P1 and 2,4,6-tris[3-(diphenylphosphinyl)phenyl]-1,3,5-triazine (PO-T2T), with a singlet-triplet splitting of 7 meV. It is noted that the contribution of harvested triplets in monochrome organic light-emitting diodes (OLEDs) correlates with *Φ*_DF_. For devices based on intermolecular CT-emission, the harvested triplets contribute ~90% to the internal quantum efficiency. The results demonstrate the vital importance of host materials on improving the PLQY and sensitizing *Φ*_DF_ of TADF polymers for efficient devices. Solution-processed polychrome OLEDs with a color close to a white emission are presented, with the emission of intramolecular (P1) and intermolecular TADF (PO-T2T:P1).

## Introduction

Since the first report of organic light-emitting diodes (OLEDs) by Tang and Vanslyke (1987), great efforts have been dedicated to achieve efficient and cost-effective OLED architectures for display and lighting applications. In the early stage of research, the device efficiency was limited by non-radiative triplets with a share of ~75% of all excitons, generated directly under electrical excitation in conventional fluorescent emitters (Segal et al., 2003). As though triplet-triplet-annihilation can generate one emissive singlet from two triplets, the theoretical limit of the internal quantum efficiency (IQE) utilizing this bimolecular pathway is limited to 62.5% (Zhang and Forrest, 2012). The development of phosphorescent emitters, generally organometallic compounds with heavy metal atoms such as iridium, palladium and gold, allows harvesting triplet excitons through phosphorescence emission, making unity IQE values possible (Thompson, 2007). Highly efficient monochrome and white OLEDs have been demonstrated with phosphorescent emitters by using optimized multilayer architectures via thermal deposition under high vacuum condition (Reineke et al., 2009, 2013; Li et al., 2017, 2018). Still, phosphorescent emitters have several drawbacks in their use in OLEDs. The use of metal ingredients, leading to concerns of environmental hazards and high costs, drives the research community to find more environmentally friendly and cost effective alternatives. Furthermore, vacuum deposition has a high energy footprint compared to solution processes such as spin-coating or printing. The development of solution processable, purely organic materials is still active both in scientific and industry fields (Zheng et al., 2013; JOLED, 2018; Wei et al., 2018a).

An alternative way to utilize triplets is converting them to singlets by finely matching the triplet and singlet state energy with a small singlet-triplet splitting Δ*E*_ST_, where reverse intersystem crossing (RISC) occurs by harvesting the environmental thermal energy, known as thermally activated delayed florescence (TADF). In 2011, the first purely organic, reliable TADF emitter PIC-TRZ was reported by Endo et al., with a moderate photoluminescence quantum yield (PLQY) of 39% and merely 32% triplet harvesting efficiency when doped in 1,3-bis(N-carbazolyl)benzene (mCP) in the device (Endo et al., 2011). Later in 2012, the same group has reported purely organic TADF emitters with almost 100% triplet harvesting efficiency (Uoyama et al., 2012). After this breakthrough, numerous efforts have been devoted to the purely organic TADF small molecules, which can be purified by sublimation and processed by physical vapor deposition to obtain multilayer devices (Uoyama et al., 2012; Dias et al., 2013; Jankus et al., 2014; Zhang et al., 2016; Wong and Zysman-Colman, 2017).

**Figure 1:**
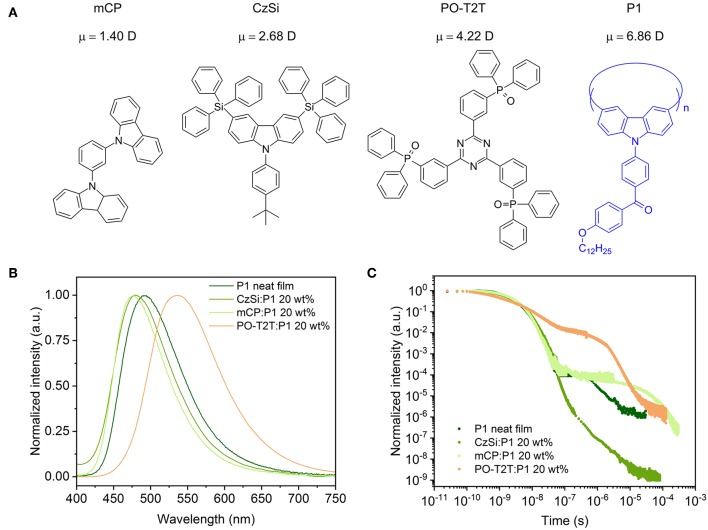
**(A)** Chemical structure of host materials and the blue TADF polymer P1, with the calculated electrical dipole moment μ for different host materials. The trimer of P1 (*n* = 3) is set as the host for the P1 neat film in dipole moment calculations. **(B)** PL spectra of different spin-coated films with P1. **(C)** Transient PL decay measured at room temperature (ca. 297 K), detected at the peak wavelength of the respective steady state PL spectrum, excited with a pulse laser at a wavelength of 373 nm.

On the other hand, TADF devices based on dendrimers and/or polymers have also gained huge attention, because of the possibility of easy fabrication by solution processes and high efficiency at the same time. As a proof-of-concept, solution-processed high efficiency OLEDs based on modified TADF small molecules with a better solubility have been reported (Cho et al., 2014). Meanwhile, to achieve macromolecules showing TADF, there are in general two approaches: (i) incorporating TADF monomers into polymer side chains with a non-conjugated backbone, and (ii) polymerizing donor and acceptor parts to form the main chain where the charge transfer state emission has TADF character, and each TADF unit is separated without conjugation (Li et al., 2016; Huang et al., 2018; Lin et al., 2018b; Wei et al., 2018b). For the first design principle, it is quite straightforward to achieve TADF macromolecules by binding the TADF monomeric units to a non-conjugated polymer side chain or linking with dendrimer groups without breaking π-conjugation among the TADF moieties (Luo et al., 2016; Xie et al., 2017; Yang et al., 2018). For the second approach, the conjugation between different TADF units are supposed to be disturbed by σ bonds. The first TADF polymer was denoted as “intermonomer TADF” based on the second strategy, and a high external quantum efficiency (EQE) of 10% was obtained (Nikolaenko et al., 2015). It should be noted that besides these two general design principles, there are other possibilities to achieve TADF polymers. Wang et al. reported solution-processed OLEDs with a maximum EQE (EQE_max_) of 12.1% based on a TADF polymer via the so-called through-space charge transfer effect (Shao et al., 2017). Previously, we reported a method to generate an efficient blue TADF polymer, denoted P1 (Figure 1), by polymerizing a non-TADF monomer (4-(3,6-dibromo-carbazol-9-yl)phenyl)(4-(dodecyloxy)phenyl)methanone to conjugated polymer macrocycles. The appearance of TADF is resulting from the conjugation-induced reduction of the effective energy splitting Δ*E*_ST_, while keeping a sufficient fast radiative decay rate (Wei et al., 2017). The previous report on P1 focused on the synthesis and the origin of the TADF characteristics, without further investigation of its device integration and, in particular, on the influence of host materials in a device surrounding.

For small molecule TADF emitters in host-guest systems, the host materials can have a significant impact on the device efficiency and lifetime (Nakanotani et al., 2013; Cui et al., 2017). Recently, Han et al. reported that the host-guest dipole interaction can influence the PLQY of a group of blue TADF small molecules. Reducing the excited-state dipole moments of host materials can slightly reduce the rate of RISC, while the non-radiative rate is significantly suppressed, leading to a large improvement of the device performance (Han et al., 2018). However, there is still a lack of understanding of the interaction between host materials and TADF polymer emitters. Furthermore, even though tremendous efforts have been put on intermolecular charge transfer state (CT-state) TADF (in the OLED community typically referred to as “exciplex”) between small molecules (Goushi et al., 2012; Liu et al., 2015; Wu et al., 2017; Lin et al., 2018a; Ullbrich et al., 2019), the first report of intermolecular CT-emission with TADF characteristics based on a non-TADF polymer (poly(9-vinylcarbazole), PVK) and small molecule (2,4,6-tris[3-(diphenylphosphinyl)phenyl]-1,3,5-triazine, PO-T2T) was recently reported by Pander et al. (2018). In their system, the concentration of small molecule component has only minor influence on the transient PL decay profile of the CT-emission.

Here, we report our detailed investigation of the interactions between different host materials and the guest TADF polymer P1. We note that the host material can induce important effects on the PLQY and TADF characteristics (time dynamics and efficiency) of the polymer emitter. In the non-doped system (neat polymer film), weak delayed fluorescence is observed with a fraction of 2.95% of the total photoluminescence (PL). In the wide-gap and high triplet level 9-(4-tert-butylphenyl)-3,6-bis(triphenylsilyl)-9H-carbazole (CzSi) host, the PLQY is reduced, and the delayed fluorescence is almost completely quenched (0.014% of the entire emission). Both the PLQY and the delayed fluorescence are significantly increased when using mCP as host material. The PLQY is increased to 49.8% for mCP:P1 (20 wt%), which is about 2.5 times higher than that for CzSi:P1 (20 wt%). Moreover, the fraction of delayed fluorescence reaches about 66% of the entire fluorescence in the mCP host. This indicates that the absolute quantum yield of delayed fluorescence (*Φ*_DF_) is enhanced by a factor of about 5,900 with mCP as host compared to CzSi. Further investigations show that P1 and PO-T2T can form an intermolecular CT-emission with substantial TADF characteristics. The emission spectrum is hardly dependent on the P1 concentration in the range of 20–60 wt%. The singlet-triplet splitting Δ*E*_ST_ for the intermolecular CT-emission is as small as 7 meV, leading to a very pronounced TADF emission, with a ratio of 2.13 between *Φ*_DF_ and the quantum yield of prompt fluorescence (*Φ*_PF_). It is estimated that in OLEDs utilizing the delayed emission, P1:PO-T2T intermolecular CT-emission contributes to about 90% to the IQE. Combining blue emission from P1 and yellow emission from the PO-T2T:P1 CT-emission, we demonstrate broadband, polychromatic OLEDs with a single emission layer, which holds promise for solution-processed white OLEDs based on the collective effect of two distinct CT-states, both giving rise to TADF.

## Results and Discussion

### Host Environment-Enhanced TADF of P1

The PLQY and the delayed fluorescence for the TADF polymer P1 are sensitive to the surrounding environment. The P1 neat film shows a moderate PLQY of 30.7%, with a PL spectrum peaking at ~490 nm. When embedded in a host material (structures shown in Figure 1A) with a wide energy gap and high triplet energy (**Figure 3A**), the PLQY for the mixed film changes. As summarized in Table 1, when P1 doped with a concentration of 20 wt% in CzSi, a widely used host material for blue emitters with a wide bandgap (Cho et al., 2014), a lowering of the PLQY (23.9%) is observed. The PLQY in CzSi is slightly increased up to ~30%, with different P1 concentration from 10 to 25 wt%, as shown in Figure S1. For mCP:P1 films, the PLQY reaches 57.7% at 10 wt% of P1. It decreases to 49.4% for an increased doping concentration of P1 of 25 wt%. The PLQY values of P1 in this study do not reach the values as initially reported previously (Wei et al., 2017), which was obtained for a different host material (polystyrene). The change of the doping concentration within the range of 10–25 wt% has only a minor influence on the PL spectrum, as shown in Figure S1. The PL spectrum of mCP:P1 (20 wt%) film peaks at 477 nm, which is slightly blue-shifted compared to the P1 neat film, beneficial for achieving blue OLEDs. CzSi is a widely used host material with photoluminescence in the UV/deep blue spectral range (Tsai et al., 2006). Furthermore, with HOMO and LUMO values of 6.0 and 2.5 eV, respectively (Baranoff and Curchod, 2015), CzSi forms a type-I hetero-interface, not allowing for a charge-transfer state to form. Thus, the blue emission of CzSi:P1 mixture can be attributed to the intrinsic P1 emission. The change of the emission spectrum of mCP, CzSi and non-doped film can be attributed to the different interaction of P1 with the various hosts having different permanent dipole moments (Reineke et al., 2010). As shown in Figure 1A, the dipole moment of the mCP ground state is as small as 1.40 D, while it is 6.86 D for P1. Here, a trimer section of P1 (*n* = 3) is assumed as the embedding material for the P1 neat film in the density functional theory (DFT) calculation in an attempt to properly model the surrounding environment. In case of the PO-T2T:P1 mixture (20 wt%), a substantial red-shift of the PL emission peak to 532 nm is observed, suggesting a different origin of this emission. Here, an intermolecular CT-emission between P1 and PO-T2T gives rise to this distinct PL. Before systematically investigating the photophysical property of the PO-T2T and P1 mixture, the detailed influence of host materials mCP and CzSi on the TADF characteristics is analyzed.

**Table 1:** Photophysical properties of thin films with P1.

**Film**	***Φ*_**PLQY**_ (%)**	**λ_**max**_ (nm)**	***Φ*_**PF**_ (%)**	***Φ*_**DF**_ (%)**	***Φ*_**DF**_/*Φ*_**PF**_**	**τ_**PF**_ (ns)**	**τ_**DF**_ (μs)**	***k*_**r**_ ( ×10^**7**^ s^**−1**^)**
P1 neat film	30.7	490	29.8	0.90	0.03	3.60	1.81	8.27
CzSi: P1 20 wt%	23.9	477	23.9	2.86 × 10^−3^	1.20 × 10^−4^	3.85	0.72	6.21
mCP: P1 20 wt%	49.8	476	32.9	16.9	0.52	3.44	44.32	9.50
PO-T2T: P1 20 wt%	8.1	532	2.6	5.5	2.13	13.00	1.21	0.20

Time-correlated single-photon-counting (TCSPC) measurements performed at the peak wavelength of the PL spectra show that the host molecule has a substantial influence on the decay profile of the prompt and delayed fluorescence. As summarized in Supplementary Note 1, the average lifetime of prompt (τ_PF_) and delayed fluorescence (τ_DF_) can be obtained by fitting the decay curves with multiple exponential functions, while the quantum yield of the prompt and delayed fluorescence *Φ*_PF_ and *Φ*_DF_ can be calculated with the weighting ratio of the integrated area of these decay curves (Lakowicz, 2006). The detailed fitting process is shown in Figure S3 and the fitting parameters are summarized in Tables S1, S2. The photophysical properties of P1 in different hosts are summarized in Table 1. The τ_PF_ is 3.60 ns for P1 neat film. It is slightly different for CzSi (3.85 ns) and mCP (3.44 ns). The τ_DF_ varies significantly for the neat and doped films. The τ_DF_ is 1.81 μs for P1 neat film, while it is 0.72 μs in CzSi and 44.3 μs in mCP. Moreover, big variations of the *Φ*_PF_, *Φ*_DF_, and further the ratio of *Φ*_PF_/*Φ*_DF_ are observed. In the neat film, only a very small amount of delayed fluorescence (*Φ*_DF_ = 0.90%) is observed, with a ratio of *Φ*_DF_/*Φ*_PF_ = 0.03. When embedding P1 in the CzSi host, the delayed fluorescence is negligible, with *Φ*_DF_ of only 0.0029%, and extremely low *Φ*_DF_/*Φ*_PF_ (0.00014). The delayed fluorescence is much more pronounced when using mCP as host material, for which *Φ*_DF_ = 16.9% and *Φ*_DF_/*Φ*_PF_ = 0.52 are obtained. Compared to the CzSi host material, the *Φ*_DF_ in mCP host is more than 5,900 times higher.

It is interesting to note that, even though the singlet state is decreasing by increasing the dipole moment of host materials (Figure 1A), the ratio *Φ*_DF_/*Φ*_PF_ is not fully following the trend. As we can see, the PL emission is red-shifted for the non-doped film compared to the mCP:P1 system, while the ratio *Φ*_DF_/*Φ*_PF_ is still much lower compared to the later. Thus, a deeper blue emission together with a higher fraction of delayed fluorescence are obtained at the same time in mCP, which is beneficial for fabricating blue OLEDs. According to the photophysical investigation and DFT analysis, factors including host-guest energy transfer, dipole moment of host materials, and CT-state generation can contribute to different *Φ*_DF_/*Φ*_PF_ ratios for the TADF polymer in different hosts.

A low value of *Φ*_DF_/*Φ*_PF_ can jeopardize the triplet harvesting in electroluminescence, since triplets are generated directly with a large fraction (75%) (Segal et al., 2003), while they are generated from singlets via intersystem crossing (ISC) under optical excitation. The extremely low value of *Φ*_DF_/*Φ*_PF_ in the CzSi host gives a hint that most of the generated triplets in the device cannot efficiently transfer to singlets, rendering this specific material combination unsuitable for device applications.

### Photophysical Properties of the PO-T2T:P1 Mixture

**Figure 2:**
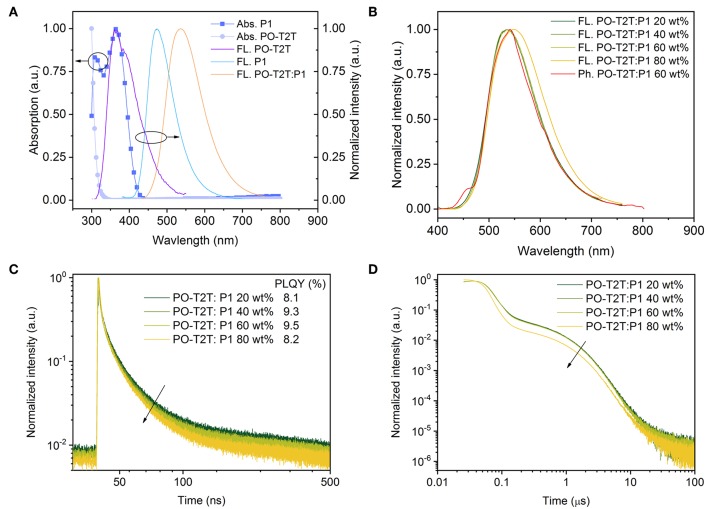
The CT-emission between PO-T2T and P1 showing thermally activated delayed fluorescence. **(A)** Normalized absorption, fluorescence spectrum of P1 and PO-T2T, and the CT-emission of the PO-T2T:P1 mixture. **(B)** Room temperature steady state fluorescence spectra of PO-T2T:P1 with different mixing ratios, compared to a representative phosphorescence spectrum of the PO-T2T:P1 60 wt% film obtained in liquid nitrogen. **(C)** Prompt fluorescence decay of the PO-T2T:P1 CT-emission. The PLQY for the various mixtures is given in the legend. **(D)** Overview of the PL decay for the different PO-T2T:P1 compositions showing prompt and delayed fluorescence (TADF). At very long times, a third channel (>10 μs) is visible, suggesting weak phosphorescence.

As mentioned above, the large shift of the PL spectrum of the PO-T2T:P1 mixed film indicates a possible intermolecular CT-emission. As shown in Figure 2A, compared to the pure emission of PO-T2T and P1, the spectrum of the mixed film is red-shifted by ~170 and 60 nm, respectively. According to previous reports, the LUMO level of PO-T2T is about 3.5 eV and the HOMO level of P1 is 5.7–5.8 eV (Wu et al., 2017). The PO-T2T as the donor and P1 as the acceptor can form a CT-state with an energy gap of about 2.2–2.3 eV, which is close to the emission energy of the PO-T2T:P1 mixture, supporting the concept that the yellow light emission in the PO-T2T:P1 blend is resulting from charge transfer between PO-T2T and P1. As shown in Figure 2B, there is no significant change of the PL spectra measured under ambient condition, with the PL peak at ~540 nm, when varying the P1 concentration in the PO-T2T and P1 mixture films from 20 to 60 wt%. Increasing the concentration of P1 to 80 wt% can slightly shift the PL maximum to 548 nm.

In the following, the emission dynamics of this PO-T2T:P1 mixture are discussed. The phosphorescence spectra measured at 77 K show only a small red-shift compared to the steady PL spectra, as shown in Figure 2B and Figure S2. The similarity of PL spectra at room temperature and the phosphorescence spectra (77 K) indicates a small Δ*E*_ST_. According to Supplementary Note 2, as shown in Figure S4 and Table S3, by fitting the fluorescence and phosphorescence spectra, the singlet energy level is 2.162 eV, while the triplet energy level is 2.155 eV, giving a splitting Δ*E*_ST_ as small as 7 meV.

Similar PL transients have been obtained for the PO-T2T:P1 mixture with varied P1 concentration from 20 to 80 wt%, as shown in Figures 2C,D. The decay time of prompt fluorescence τ_PF_ is 13.0 ns for 20 wt% P1, while it only slightly decreases to 11.1, 11.2, and 8.6 ns, for 40, 60, and 80 wt% P1 films, respectively. The decay time of delayed fluorescence τ_DF_ is around 1.2 μs, with a minor variation with different P1 concentrations from 20 to 60 wt%. A more pronounced change for the PO-T2T:P1 (80 wt%) film goes hand in hand with the observed red-shift of steady state PL emission, as shown in Figure 2B. Our results show that the ratio between donor and acceptor has minor influence on the transient decay of the CT-emission, which is similar compared to the first reported CT-emission with TADF characteristics between small molecules and polymers (Pander et al., 2018).

The PLQY of the PO-T2T:P1 system is about 8–10% (Figure 2C), demonstrating that non-radiative decay dominates the relaxation process. Nevertheless, a significant delayed fluorescence is observed from the CT-emission, with a ratio of *Φ*_DF_/*Φ*_PF_ larger than 2, indicating that there are cycling processes from triplets and singlets (Wei et al., 2017). The detailed photophysical properties of PO-T2T:P1 (20 wt%) are also summarized in Table 1.

### Monochrome OLEDs Based on P1

**Figure 3:**
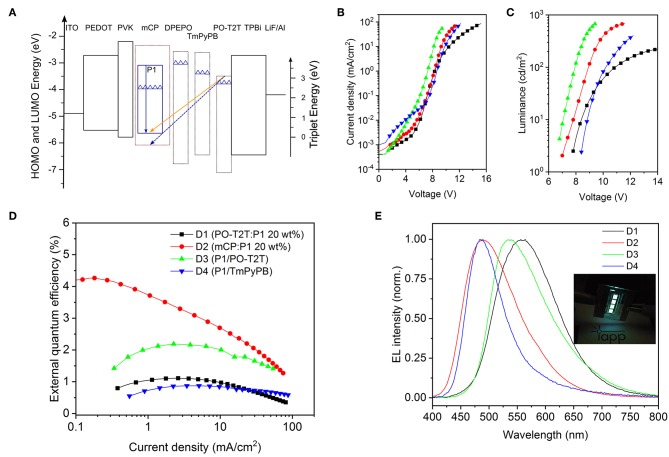
Monochrome OLEDs based on P1. **(A)** Schematic diagram of energy levels of representative devices (Su et al., 2008; Wu et al., 2017). The top line of each box indicates the LUMO, and the bottom line refers to the HOMO level. The solid blue arrow represents the intrinsic emission from the TADF polymer P1, while the orange arrow represents the CT-emission between PO-T2T and P1, and the dashed blue line represents the possible CT-emission between host mCP and PO-T2T (Liu et al., 2015; Wu et al., 2017). The triangles refer to the triplet energy levels (Su et al., 2008; Wu et al., 2017). **(B)** Voltage-current density characteristics, **(C)** voltage-luminance characteristics, and **(D)** current density-external quantum efficiency dependencies of monochrome devices. Panels **B–D** share the same legend. **(E)** Respective normalized EL spectra at 100 cd/m^2^. The inset picture is a photo of the OLEDs with mCP as host material.

Based on the photophysical investigations, we further explore monochrome OLEDs based on P1 with different device structures. The device structures and characteristics are summarized in Table 2. The energy diagram is shown in Figure 3A (Su et al., 2008; Wu et al., 2017). As depicted in Figure 3B, for the monochrome devices D1 (PO-T2T:P1 [20 wt%]) and D2 (mCP:P1 [20 wt%]), the slight difference of the voltage-current density characteristics stems from the different transport properties of host materials. However, a large difference of voltage-current density behavior is noted for D3 (P1/PO-T2T) and D4 (P1/1,3,5-tri(m-pyridin-3-ylphenyl)benzene, TmPyPB), as shown in Figure 3B. The reason can be ascribed to the difference between the LUMO levels for PO-T2T and TmPyPB (Figure 3A). As shown in Figure 3C, a voltage larger than 6 V is needed to turn D1–D4 on. Many reasons may contribute to the high turn-on voltage of D1 and D2, including limited transport mobility of the PVK and PEDOT:PSS layer (Pander et al., 2018).

**Table 2:** Summary of the device characteristics.

**Device**	**Device structure^a^ (EBL/EML/HBL/ETL/EIL)**	**EQE_**max**_ (%)**	**CE_**max**_ (cd/A)**	**LE_**max**_ (lm/W)**	**CIE (x, y)^b^**
D1	PVK (15 nm)/PO-T2T:P1 20 wt% (50 nm)/DPEPO (10 nm)/TPBi (50 nm)/LiF (1 nm)	1.1	0.9	0.9	(0.42, 0.52)
D2	PVK (15 nm)/mCP:P1 20 wt% (50 nm)/DPEPO (10 nm)/TPBi (50 nm)/LiF (1 nm)	4.3	3.0	2.5	(0.24, 0.37)
D3	P1 (30 nm)/PO-T2T (10 nm)/Bphen:Cs (50 nm)	2.2	1.9	1.9	(0.39, 0.54)
D4	P1 (30 nm)/TmPyPB (10 nm)/Bphen:Cs (50 nm)	0.9	0.7	0.4	(0.20, 0.35)
D5	PVK (15 nm)/PO-T2T:P1 99 wt% (50 nm)/DPEPO (10 nm)/TPBi (50 nm)/LiF (1 nm)	1.7	1.8	1.8	(0.28, 0.40)
D6	PVK (15 nm)/PO-T2T:P1 99.5 wt% (50 nm)/DPEPO (10 nm)/TPBi (50 nm)/LiF (1 nm)	1.2	1.3	1.6	(0.31, 0.44)

a *The complete device is composed of ITO/PEDOT:PSS (70 nm)/EBL/EML/HBL/EIL/Al (100 nm). For D3 and D4, no EBL, and EIL are used*.

b *D1–D4 are obtained at a driving current of 0.5 mA, while D5-D6 are obtained at 100 cd/m^2^*.

For D4 with P1 neat film as emitting layer, as shown in Figure 3D and Table 2, a quite low EQE_max_ of 0.87% is obtained. The reason could be an imbalanced charge carrier injection, an intrinsically lower PLQY and/or an inefficient RISC process. A further device optimization may enhance the charge balance and triplet diffusion to the hole transport layer. A medium EQE_max_ of 4.26% can be obtained for D2 with 20 wt% P1 doped in mCP, as shown in Figure 3D and Table 2. The EQE_max_ is 1.11% for D1 and 2.19% for D3, both showing the CT-emission between PO-T2T and P1.

As shown in Figure 3E and Table 2, the emission color for D2 and D4 is cyan blue, with the Commission Internationale de l'Eclairage (CIE) coordinates (0.24, 0.37) for D2 and (0.20, 0.35) for D4. However, a substantially different emission color is noted for D1 (PO-T2T:20 wt% P1) and D3 (P1/PO-T2T). The EL spectra with a peak wavelength located at about 530 nm for D3 is very close to the PL spectrum of the CT-emission between PO-T2T and P1 (Figure 2B). The device cavity and the emission from intrinsic P1 may contribute to a slight deviation of electroluminescence for D1 from the PL spectrum of the PO-T2T:P1 CT-state.

### Triplet Harvesting in Monochrome OLEDs

Under electroluminescence in OLEDs, 75% of the excitons are generated as triplets in the first place (Segal et al., 2003). Hence, if there is a lack of efficient RISC within the TADF system, the triplets cannot fully transfer to emissive singlets. Without the consideration of bimolecular annihilation processes, the EQE is determined by the following equation (Li et al., 2018):

(1) $$\begin{array}{l} \text{EQE}=\gamma \eta_{\text{int}}\eta_{\text{out}} \end{array}$$

where γ is the electrical efficiency, η_int_ is the IQE and η_out_ is the outcoupling efficiency. For OLEDs based on TADF emitter, η_int_ can be calculated as (Endo et al., 2011; Goushi et al., 2012):

(2) $$\begin{array}{l} \eta_{\text{int}}=0.25\phi_{\text{PF}}+\sum_{i=1}^{\infty }0.25\phi_{\text{PF}}{\left(\phi_{\text{ISC}}\phi_{\text{RISC}}\right)}^{i}\\ \;+\;\sum_{i=0}^{\infty }0.75\phi_{\text{PF}}{\phi_{\text{RISC}}\left(\phi_{\text{ISC}}\phi_{\text{RISC}}\right)}^{i} \end{array}$$

where *Φ*_ISC_ indicates the quantum yield of ISC, while *Φ*_RISC_ is the quantum yield of RISC. The term of *Φ*_ISC_*Φ*_RISC_ indicates the cycling process *i* from singlets to triplets. The contribution of the prompt fluorescence η_int_PF_ can be calculated as (Goushi et al., 2012; Lee et al., 2013):

(3) $$\begin{array}{l} \eta_{\text{int\_PF}}=0.25\phi_{\text{PF}} \end{array}$$

The final mathematical form of Equation (2) can be written as:

(4) $$\begin{array}{l} \eta_{\text{int}}=\phi_{\text{PF}}\;\left(\frac{0.25}{1-\phi_{\text{ISC}}\phi_{\text{RISC}}}+\frac{0.75}{1-\phi_{\text{ISC}}\phi_{\text{RISC}}}\phi_{RISC}\right) \end{array}$$

Kinetically, for an efficient TADF emitter with a predominant delayed emission, the non-radiative rate of the singlet is of similar magnitude as the radiative rate of the singlet, in the range of 10^8^ s^−1^. For the triplets, the non-radiative rate should be comparable to the rate of reverse intersystem crossing, which is normally lower than 10^6^ s^−1^ (Dias et al., 2016, 2017). Since the non-radiative rate of singlets is orders higher than the non-radiative rate of triplets, we assume that the non-radiative relaxation comes merely from the singlets state. Under this assumption, *Φ*_RISC_ = 1, while *Φ*_ISC_ can be calculated as (Dias et al., 2017):

(5) $$\begin{array}{l} \phi_{\text{ISC}}=\frac{\phi_{\text{DF}}}{\phi_{\text{DF}}+\phi_{\text{PF}}} \end{array}$$

With Equation (5), η_int_ in form of Equation (4) can be calculated with *Φ*_DF_ and *Φ*_PF_:

(6) $$\begin{array}{l} \eta_{\text{int}}=\phi_{\text{DF}}+\phi_{\text{PF}} \end{array}$$

The contribution of delayed fluorescence η_int_DF_ or the quantum efficiency from harvesting triplets, can be then calculated from:

(7) $$\begin{array}{l} \eta_{\text{int\_DF}}=\eta_{\text{int}}-0.25\phi_{\text{PF}}=\phi_{\text{DF}}+{0.75\phi }_{\text{PF}} \end{array}$$

**Figure 4:**
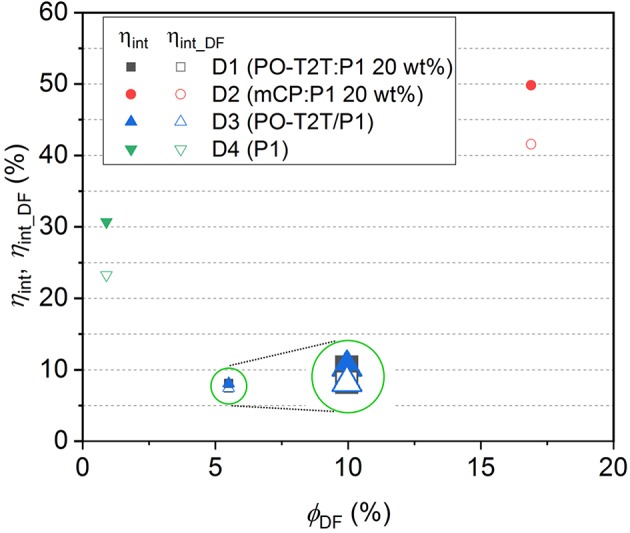
The quantum efficiency in device D1–D4. The inset circles indicate a schematic magnification for D1 and D3, for which both the internal quantum efficiency and η_int_DF_ are overlapping with each other.

Based on Equations (6) and (7), η_int_ and η_int_DF_ for devices D1–D4 can be calculated, as summarized in Table 3. The dependency of the η_int_ and η_int_DF_ on *Φ*_DF_ for D1–D4 is shown in Figure 4. Since η_int_ depends on the PLQY of the emitting layer, the η_int_ of D2 with mCP:P1 as the emissive layer is 49.8%, which is about 6 times higher compared to D1 and D3 based on the PO-T2T:P1 mixture and 1.6 times higher than the D4 with P1 neat film. For devices based on a P1 neat film, the η_int_ remains 30.7%, but the delayed fluorescence contributes only 76% to the IQE. For the PO-T2T:P1 emission system, even though η_int_ for D1 and D3 is only 8.1%, the η_int_DF_ contributes 92% to η_int_, which can be assigned to the predominant delayed fluorescence with *Φ*_DF_/*Φ*_PF_ as high as 2.13. Since *Φ*_DF_/*Φ*_PF_ for mCP:P1 is slightly lower compared to PO-T2T:P1 mixture, the delayed emission contributes ~83% among η_int_ in D2.

**Table 3:** Summary of device performance of monochrome OLEDs.

**Device**	**EML structure**	**η_**int**_**	**η_**int_DF**_**	**η_**int_DF**_/η_**int**_**	**η_**out**_**	**γ^d^**
		**(%)^a^**	**(%)^b^**		**(%)^c^**	
D1	PO-T2T:P1 20 wt%	8.1	7.4	0.92	15.3	0.88
D2	mCP:P1 20 wt%	49.8	41.6	0.83	12.3	0.69
D3	PO-T2T/P1	8.1	7.4	0.92	22.2	1.22
D4	P1 neat film	30.7	23.2	0.76	21.1	0.13

a *Calculated from Equation (6)*.

b *Calculated from Equation (7)*.

c *Simulation results. Details in experimental sections*.

d *Electrical efficiency, calculated from Equation (1)*.

The outcoupling efficiency η_out_ can be simulated by transfer matrix method (details in the experimental section), and the results are summarized in Table 3. The electrical efficiency corresponds to the charge balance and recombination. For state-of-the-art thermal deposited OLEDs, the electrical efficiency γ is around 0.8–1.0 (Furno et al., 2012). The electrical efficiency for each device can be calculated according to Equation (1) based on η_out_ and η_int_ with the EQE_max_. As shown in Table 3, the electrical efficiency γ for D1 and D2 is 0.88 and 0.69, while it is 1.22 for D3, which is physically not meaningful. One possible reason could be the improper assumption of an isotropic emitter and the emission from intrinsic P1. Nevertheless, the electrical efficiency for D4 is only 0.13, indicating that the electrical loss also contributes to the low device efficiency. Further optimization of D4 may give a slightly higher efficiency. The HOMO and LUMO level of the host materials can influence the charge carrier injection barriers from the adjacent layers with direct influence on the electrical efficiency through altered charge injection and/or charge recombination. Various host materials with different HOMO and LUMO levels in D1–D4, as shown in Figure 3A, can be one of the possible reasons for the difference of the estimated electrical efficiency.

It should be noted that the triplets can also give non-radiative relaxation for the TADF emitters, leading an over-estimation of the contribution of harvested triplets to the IQE. Even though the real η_int_DF_ and η_int_ for D1–D4 may be varied slightly, it is clear that higher IQE can be obtained for OLEDs based on emissive layer with a high *Φ*_DF_ and *Φ*_PF_. Therefore, the suppression of non-radiative relaxation for TADF systems from either singlets or triplets is of vital importance to achieve efficient OLEDs. On the other hand, the ratio *Φ*_DF_/*Φ*_PF_ can largely affect the contribution of the delayed emission among the total quantum efficiency in a device.

### Polychrome OLEDs With Dual Emission From P1 and CT-State

**Figure 5:**
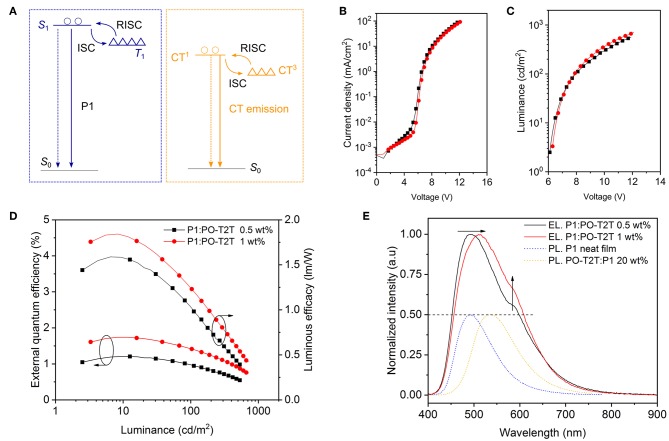
Single layer OLEDs with dual emission from the intramolecular CT of P1 and the intermolecular CT-state between P1 and PO-T2T (D5 and D6 of Table 2). **(A)** Simplified energy level diagram for the mixed film comprising P1 and PO-T2T. The solid lines represent the radiative relaxation, while dashed lines represent the non-radiative loss. **(B)** The voltage-current density, **(C)** the voltage-luminance, and EQE-luminance-luminous efficacy characteristics of D5 and D6. **(B–D)** share the same legend. **(E)** Normalized EL spectra for D5 and D6 in the forward direction obtained at 100 cd/m^2^. The dotted PL spectra of P1 and the P1:PO-T2T CT state are shown for comparison and normalized to 0.5. The dashed line represents the FWHM of the EL spectrum. The arrows indicate that the fraction of yellow part from the intermolecular CT-emission is increasing for higher concentration of PO-T2T in the emission layer.

Polychrome OLEDs where the emission in general originates from more than one luminescent species are constructed here with a single emitting layer combining the blue emission from P1 and the yellow CT-emission between PO-T2T and P1. As shown in Figure 5A, in such a mixed film, there are two TADF processes: (i) TADF governing the luminescence of P1 itself and (ii) the CT-emission between PO-T2T and P1, which also shows TADF characteristics, as discussed above. Excitons generated in P1 can transfer to the CT-state by several processes: Förster resonance or Dexter energy transfer determined by the exciplex concentration (Higuchi et al., 2015). Here, the acceptor state is the PO-T2T:P1 CT-state characterized with—compared to local transitions—weak oscillator strength that ultimately defines the strength of these energy transfer pathways (Ullbrich et al., 2019). Additionally, the CT-state can be populated by charge separation from local donor (P1) to CT excitons as occurring in solar cells as the initial step of charge separation (Ullbrich et al., 2019). Of course, direct CT exciton formation at the interface between P1 and PO-T2T is possible. Since the increase of PO-T2T concentration in the mixed film can tune the emission to yellow (e.g., D1), the major part of the film should be P1 to achieve a double-color emission. Based on these prerequisites, we tested the P1 polymer film embedded with 0.5 wt% (D6) and 1 wt% PO-T2T (D5) to demonstrate double-color emission close to white spectrum in a single emission layer architecture.

As shown in Figure 5B and Table 2, the slight change of the PO-T2T concentration has little influence on the electrical performance of D5 and D6. The luminance for D5 is slightly higher than D6, as shown in the Figure 5C. Maximum EQEs of 1.74 and 1.20% are achieved for D5 and D6 at around 10 cd/m^2^, respectively, as shown in Figure 5D. The EQE slightly rolls off to 1.41% at 100 cd/m^2^ for D5 and 0.97% at 100 cd/m^2^ for D6. These devices show maximum luminous efficacy values of 1.84 lm/W for D5 and 1.59 lm/W for D6.

The EL spectra are shown in Figure 5E. The device D6 with 0.5 wt% PO-T2T shows two separate peaks, located at ~ 490 and 570 nm, giving a CIE of (0.31, 0.43) with a full width at half maximum (FWHM) of 146 nm. Slightly increasing the concentration of PO-T2T to 1 wt% can shift the first peak to about 510 nm, while the intensity of the shoulder peak is enhanced. In the end, D5 gives a final CIE of (0.28, 0.40) with a FWHM of 153 nm. In this architecture, constructed from a combination of a TADF polymer and a small molecule host, two different TADF mechanisms are brought together to collectively achieve broadband emission (~ 150 nm FWHM). These results indicate its potential to reach both high IQE and white light emission.

## Conclusion

In this work, we report the substantial influence of host materials on the photophysical properties of a TADF polymer P1 and further, the triplet harvesting ability in OLEDs comprising this emitter. Almost no delayed fluorescence is observed in the neat film or in the host CzSi. The delayed fluorescence can be enhanced by more than three orders of magnitude when replacing the host material CzSi by mCP. Furthermore, we observe a CT-emission between the PO-T2T and P1, which shows substantial TADF in line with a Δ*E*_ST_ as small as 7 meV and a ratio of 2.13 between delayed fluorescence to prompt fluorescence. When using the TADF polymer P1 to build monochrome OLEDs, cyan blue devices can be achieved for a P1 neat film, or for P1 embedded in mCP. A maximum EQE of 4.26% is achieved for devices with mCP as the host material, while it is only 0.87% for the device based on the P1 neat film. This demonstrates that in the device with mCP, an IQE of ~50% can be obtained with the delayed emission contributing ~76%. For devices based on a PO-T2T:P1 mixture, the delayed emission contributes ~90% to the IQE. The results clearly demonstrate the vital importance of host materials on sensitizing the delayed fluorescence in TADF polymers for efficient OLEDs.

Together with the yellow CT-emission from PO-T2T:P1 and the intrinsic blue emission from P1, OLEDs with polychromatic emission can be realized for a low doping concentration of PO-T2T. A maximum EQE of 1.74% and a luminous efficacy of 1.84 lm/W are achieved. This concept for polychrome OLEDs based on two distinct TADF routes, where one is a CT-emission between a TADF polymer and a small molecule host, can be a starting point to develop high efficiency white OLEDs based on solution processes with purely organic TADF polymers.

### Experimental

#### Materials

The PEDOT:PSS (AI4083, Heraeus Clevios™) is filtered before spin-coating. The other organic materials, 1,3-bis(N-carbazolyl)benzene (mCP, Lumtec), 9-(4-tert-butylphenyl)-3,6-bis(triphenylsilyl)-9H-carbazole (CzSi, Lumtec), 2,4,6-tris[3-(diphenylphosphinyl)phenyl]-1,3,5-triazine (PO-T2T, Lumtec), bis[2-(diphenylphosphino)phenyl] ether oxide (DPEPO, Lumtec), 4,7-diphenyl-1,10-phenanthroline (BPhen, Lumtec), 2,2′2″-(1,3,5-benzenetriyl)-tris[1-phenyl-1H-benzimidazole] (TPBi, Lumtec) and 1,3,5-tri(m-pyridin-3-ylphenyl)benzene (TmPyPB, Lumtec) are sublimated before deposition. poly(9-vinylcarbazole) (PVK, Mw ~1,100,000, Sigma-Aldrich), lithium fluoride and aluminum are used as they are purchased.

#### Photophysical Properties

Quartz substrates are cleaned with isopropanol, acetone and DI water. After heating at 110°C in an oven, the substrates are then treated with oxygen plasma for 10 min. The host–guest films are prepared by spin-coating. The film with CzSi as host is annealed at 100°C for 20 min while the mCP film is annealed at 40°C for 20 min, because of the low glass transition temperature of mCP. For transient measurements of PL emission, the TCSPC technique is used. After exciting the sample with a laser at 373 nm with pulse width of 44 ps, the emitted photons are collected by a photomultiplier tube (PicoQuant PMA Hybrid) and the data acquisition is done by a TCSPC module (PicoQuant TimeHarp 260). The PLQY of these films is confirmed by using a calibrated integrating sphere in nitrogen atmosphere, with a CAS 140 CT spectrometer and a UV-LED (Thorlabs, 340 nm). The steady-state PL spectra for P1 neat film, doped film within mCP and CzSi are collected during the PLQY measurement and used for the evaluation (Mello et al., 1997). The detailed steady-state PL spectra for the CT-emission are obtained with a Spex FluoroMax spectrofluorometer. The UV-absorption measurement is done in toluene solution, with Shimadzu MPC 3100.

#### Dipole Moment Calculation

The calculation is based on density functional theory applied to molecules in the ground state, using the functional B3LYP and a 6–31 g(d) basis set. The dipole moment for P1 is calculated on the trimer.

#### Thickness Calibration

The thickness of spin-coated films is confirmed by a profilometer (Veeco Dektak 150) and cross-checked by an imaging ellipsometer (EP4, Accurion GmbH).

#### Device Fabrication

The PEDOT:PSS solutions are filtered, spin-coated with a speed of 1,000 rpm, and then annealed at 120°C for 30 min in ambient atmosphere. The following spin-coating processes are done inside a glovebox with oxygen and water concentration lower than 1 ppm. The PVK in 1,2-dichlorobenzene with a concentration of 10 mg/ml is coated with a speed of 2,000 rpm on top of PEDOT:PSS. Before casting the emissive layer, the film is annealed at 150°C for 10 min and cooled down to room temperature. The P1 neat film has a thickness of about 30 nm by spin-coating at 1,000 rpm with a concentration of 5 mg/ml in toluene. For host-guest doped system, films with a thickness of about 50 nm can be obtained by spin-coating at a speed of 1,000 rpm with a solution concentration of 7.5 mg/ml in toluene. The emissive materials are dissolved in toluene and spin-coated on the PVK layer, with a post-annealing at 40°C for 20 min. The following organic layers are fabricated in facilities from Kurt J. Lesker Co., under the vacuum of about 10^−7^ to 10^−8^ mbar. The deposition rates for the organic materials are about 1 Å/s, detected during material evaporation through calibrated quartz crystals. The devices are then encapsulated in a glovebox before the device characterization. A finely structured mask is used for doped layers to reduce the leakage current.

#### Device Characterization

The current-voltage characteristics are measured by a Source Measure Unit (Keithley 2400), and luminance is measured simultaneously through a calibrated photodiode. The spectral radiant intensity is recorded via a calibrated spectrometer (CAS140, Instrument Systems GmbH). EQE and luminous efficacy are further measured by a calibrated integrating sphere. The active area size is 6.49 mm^2^.

#### Outcoupling Efficiency Simulation

The simulation is based on a transfer matrix algorithm, where the theory is summarized in reference (Furno et al., 2012). The anisotropy factor of the emitters for these devices is set to 0.33, corresponding to an isotropic distribution. Regarding the spectra in the optical simulation, the PL spectrum of CT-emission is used for D1 and D3, while the PL spectrum of mCP:P1 20 wt% is used for D2 and the PL spectrum of P1 neat film is used for D4. The refractive index and extinction coefficient of TPBi and PEDOT:PSS are set as measured results, shown in Figure S5. For other organic materials, the refractive index is set to 1.7, while the extinction coefficient is vanishing. The emission location is set at the interface between the emission layer and the hole blocking layer.

## Data Availability Statement

All datasets generated for this study are included in the article/Supplementary Files.

## Author Contributions

YL did the photophysical measurements and device investigations. The polymer synthesis is done by QW, LC, BV, and ZG. The dipole moment simulation is done by MC. FF contributed to the PLQY setup building and the data analysis code. YL and RS did the line shape analysis. YL, SL, and SR analyzed the device data. All the authors commented the manuscript. SL and SR organized the entire project.

### Conflict of Interest

The authors declare that the research was conducted in the absence of any commercial or financial relationships that could be construed as a potential conflict of interest.

## Supplementary Material

The supporting information includes the PL spectra for P1 doped in mCP and CzSi; the phosphorescence spectra of PO-T2T and P1 in different concentrations; The photophysical modeling for the TADF processes; decay profile fitting and lineshape fitting; refractive index.
