# Supplementary material for: Organic Light-Emitting Diodes Based on Conjugation-Induced Thermally Activated Delayed Fluorescence Polymers: Interplay Between Intra- and Intermolecular Charge Transfer States
Source: Front Chem. 2019 Oct 23;7:688. doi: 10.3389/fchem.2019.00688 (PMC6819504; doi:10.3389/fchem.2019.00688)
Supplement: Supplementary file 1 [file Data_Sheet_1.docx]

Supplementary Material

Organic light-emitting diodes based on conjugation-induced thermally activated delayed fluorescence polymers: Interplay between intra- and intermolecular charge transfer states

Yungui Li,^1*^ Qiang Wei,^2*^ Liang Cao^2,4^ Felix Fries,^1^ Matteo Cucchi,^1^ Zhongbin Wu,^1^ Reinhard Scholz,^1^ Simone Lenk,^1^ Brigitte Voit^3,5^, Ziyi Ge^2*^ and Sebastian Reineke^1*^

^1^Dresden Integrated Center for Applied Physics and Photonic Materials (IAPP) and Institute for Applied Physics, Nöthnitzer Str. 61, Technische Universität Dresden, 01187 Dresden, Germany

^2^Ningbo Institute of Materials Technology & Engineering, Chinese Academy Sciences, Ningbo 315201, P. R. China

^3^Leibniz-Institut für Polymerforschung Dresden e.V, Hohe Straße 6, 01069 Dresden, Germany

^4^Key Laboratory of Advanced Textile Materials and Manufacturing Technology, Ministry of Education, Zhejiang Sci-Tech University, Hangzhou 310018, P.R. China

^5^Organic Chemistry of Polymers, Mommsenstrasse 4, Technische Universität Dresden, 01069 Dresden, Germany

*** Correspondence:**Yungui Li: yungui.li@iapp.de

Sebastian Reineke: sebastian.reineke@tu-dresden.de

Qiang Wei: weiqiang@nimte.ac.cn

Ziyi Ge: geziyi@nimte.ac.cn


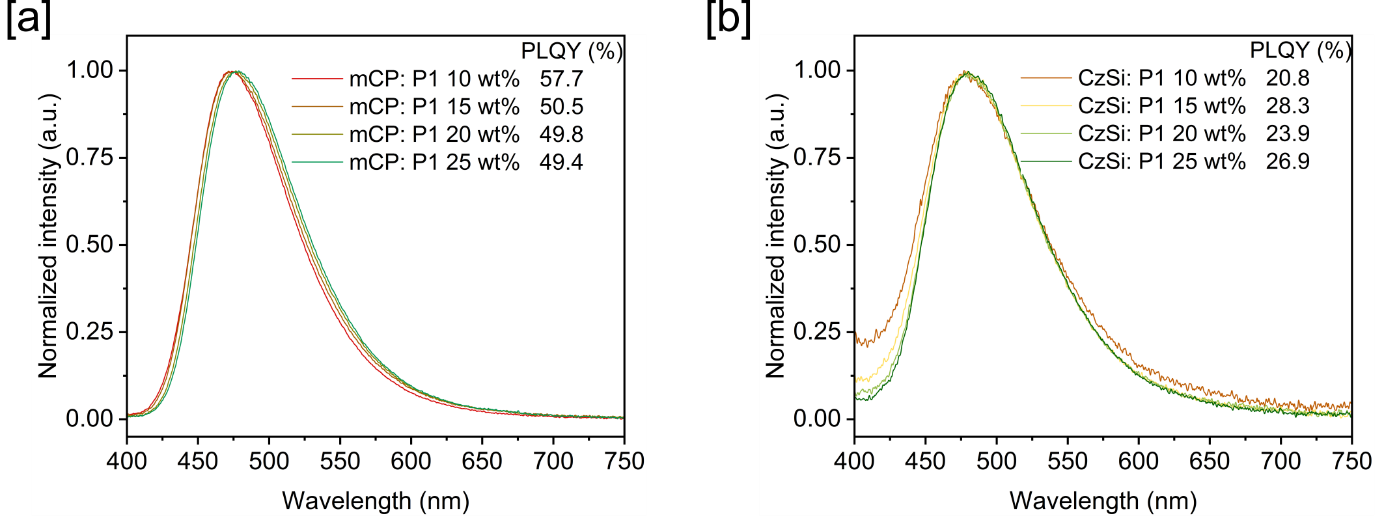


**Figure S1.** The PL spectra and the PLQY for P1 embedded in mCP (a) and in CzSi (b), with different doping concentrations and annealing recipe. mCP host films: 40 °C for 20 min; CzSi host films: 100 °C for 20 min in a glovebox.


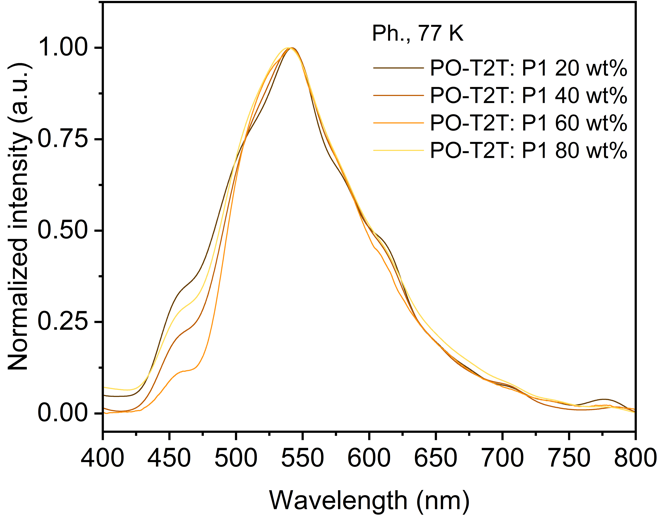


**Figure S2.** Phosphorescent spectra of PO-T2T:P1 films in liquid nitrogen (77 K).

Supplementary note 1: photophysical modeling

The decay of the prompt and delayed fluorescence can be fitted by multi-exponential functions, with:^[1]^

$$\begin{aligned} y=\sum_{i} A_{i}\exp\left( -\frac{x}{t_{i}} \right)\# \left( S1 \right) \end{aligned}$$

The averaged decay time can be obtained by:

$$\begin{aligned} \tau_{a}=\frac{\sum_{i} A_{i}t_{i}^{2}}{\sum_{i} A_{i}t_{i}}\# \left( S2 \right) \end{aligned}$$

The intensity of prompt fluorescence *I*_PF_ and delayed fluorescence *I*_DF_ can be obtained by integrating the area of transient PL measurement shown in Figure 2. The quantum yield of prompt fluorescence (Φ_PF_) and delayed fluorescence (Φ_DF_) among the total PLQY can be calculated by:^[2]^

$$\begin{aligned} \Phi_{\mathrm{PF}}=\frac{I_{\mathrm{PF}}}{I_{\mathrm{PF}}+I_{\mathrm{DF}}}\Phi_{\mathrm{PLQY}}\# \left( S3 \right) \end{aligned}$$

$$\begin{aligned} \Phi_{\mathrm{DF}}=\frac{I_{\mathrm{DF}}}{I_{\mathrm{PF}}+I_{\mathrm{DF}}}\Phi_{\mathrm{PLQY}}\# \left( S4 \right) \end{aligned}$$

Since$k_{r}\gg k_{\mathrm{RISC}}$,^[3]^ then

$$\begin{aligned} \Phi_{\mathrm{PF}}=\frac{k_{r}}{k_{r}+k_{\mathrm{nr}}^{s}+k_{\mathrm{ISC}}}=k_{r}\tau_{\mathrm{PF}}\# \left( S5 \right) \end{aligned}$$

giving

$$\begin{aligned} k_{r}=\frac{\Phi_{\mathrm{PF}}}{\tau_{\mathrm{PF}}}\# \left( S6 \right) \end{aligned}$$

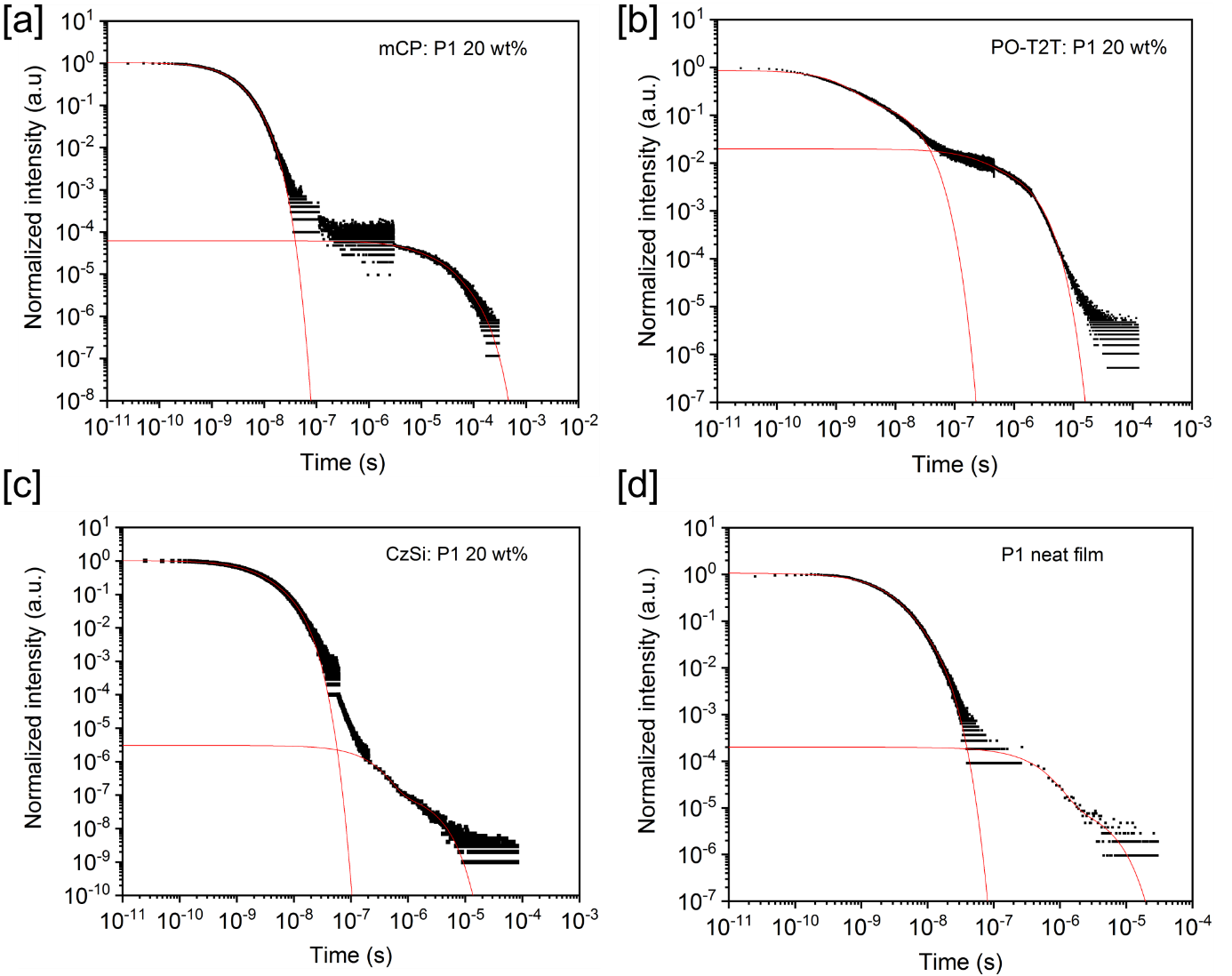


Figure S3. Transient PL decay profile fitting, measured at the peak wavelength for each film under the excitation of 375 nm laser. The red lines are fit curves. (a) mCP:P1 20 wt%, (b) PO-T2T:P1 20 wt%, (c) CzSi:P1 20 wt% and (d) P1 neat film.

Table S1. Fitting details of the prompt fluorescence and intensity (*I*_PF_).

|  | *A*_1_ | *t*_1_ (s) | *A*_2_ | *t*_2_ (s) | *τ*_PF_ (s) | *I*_PF_ |
| --- | --- | --- | --- | --- | --- | --- |
| mCP:P1 20 wt% | 0.63 | 2.04×10^-9^ | 0.4 | 4.45×10^-9^ | 3.44×10^-9^ | 3.09×10^-9^ |
| PO-T2T:P1 20 wt% | 0.66 | 1.22×10^-9^ | 0.21 | 1.58×10^-9^ | 1.30×10^-8^ | 4.6×10^-9^ |
| CzSi: P1 20 wt% | 0.53 | 1.46×10^-9^ | 0.49 | 4.66×10^-9^ | 3.85×10^-9^ | 3.09×10^-9^ |
| P1 neat film | 0.81 | 1.98×10^-9^ | 0.25 | 5.49×10^-9^ | 3.60×10^-9^ | 2.97×10^-9^ |

Table S2. Fitting details of the delayed fluorescence and intensity (*I*_DF_)

|  | *A*_1_ | *t*_1_ | *A*_2_ | *t*_2_ | *A*_3_ | *t*_3_ | *τ*_DF_ (s) | *I*_DF_ |
| --- | --- | --- | --- | --- | --- | --- | --- | --- |
| mCP: P1 20 wt% | 1.26×10^-5^ | 6.48×10^-5^ | 3.04×10^-5^ | 2.12×10^-5^ | 1.88×10^-5^ | 4.20×10^-5^ | 4.43×10^-5^ | 1.62×10^-9^ |
| PO-T2T: P1 20 wt% | 0.01015 | 2.22×10^-7^ | 0.00974 | 1.38×10^-6^ | 0 | 0 | 1.21×10^-6^ | 9.8×10^-9^ |
| CzSi: P1 20 wt% | 2.93×10^-6^ | 1.75×10^-7^ | 1.22×10^-7^ | 1.92×10^-6^ | 0 | 0 | 7.21×10^-7^ | 4.25×10^-13^ |
| P1 neat film | 1.88×10^-4^ | 4.26×10^-7^ | 1.22×10^-5^ | 4.05×10^-6^ | 0 | 0 | 1.81×10^-6^ | 9.02×10^-11^ |

Supplementary note 2: lineshape fitting

Based on the PL spectra in Figure 2, a Gaussian lineshape fitting was conducted to determine the vertical transition energies *E*_vert_ for the CT-emission. The singlet and triplet energy levels were estimated from *E*_vert_ and consequently ∆*E*_ST_. Figure S4 contains the fit curve and Table S3 summarizes the parameters gained from the line shape analysis.^[4]^

$$\begin{aligned} \frac{I\left( E \right)}{E^{3}}=a_{1}\sum_{n=0}^{\infty} \left( \frac{s^{n}}{n!}e^{-s} \right)G_{n}\left( x,\sigma_{n},E_{\mathrm{vert}}, E_{\mathrm{vibr}} \right) \\ =a_{1}\sum_{n=0}^{\infty} \left( \frac{s^{n}}{n!}e^{-s} \right)\left[ \exp\left( -\frac{\left( x-\left( E_{\mathrm{vert}}+s*E_{\mathrm{vibr}}-n*E_{\mathrm{vibr}} \right) \right)^{2}}{2\sigma_{n}^{2}} \right) \frac{1}{\sigma_{n}\sqrt{2\pi}} \right]\# \left( S7 \right) \end{aligned}$$

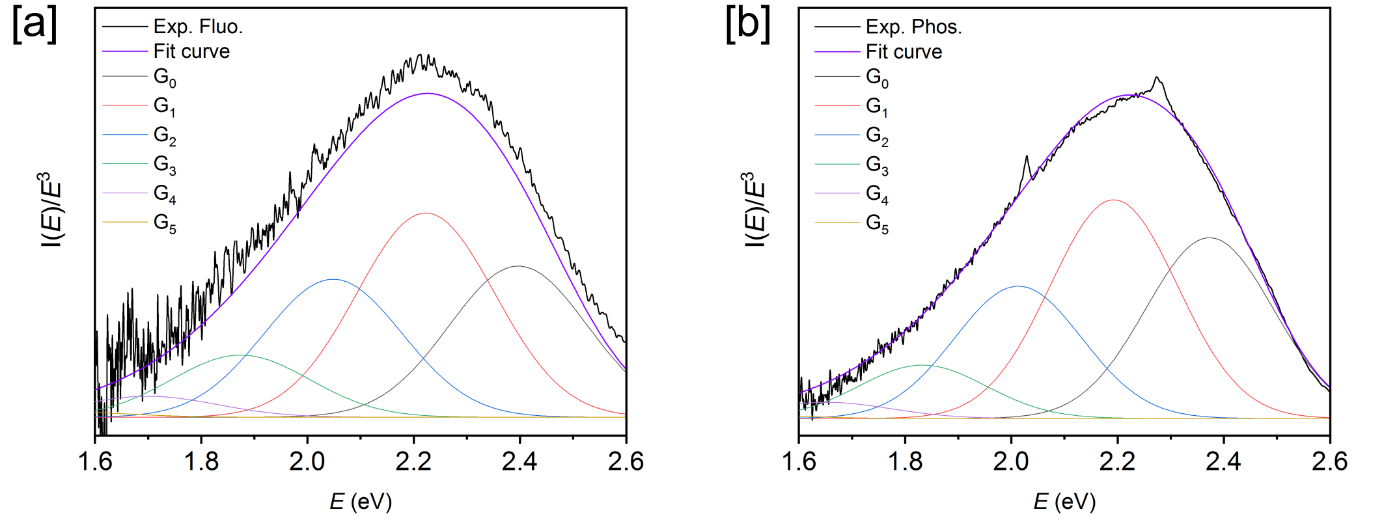


Figure S4. Lineshape fitting of CT-emission. (a) fluorescence (b) phosphorescence.

Table S3. Lineshape fitting parameters.

|  | *s* | *E*_vibr_ (eV) | *E*_vert_ (eV) |
| --- | --- | --- | --- |
| Fluo. | 1.35061 | 0.174 | 2.162 |
| Phos. | 1.20916 | 0.180 | 2.155 |


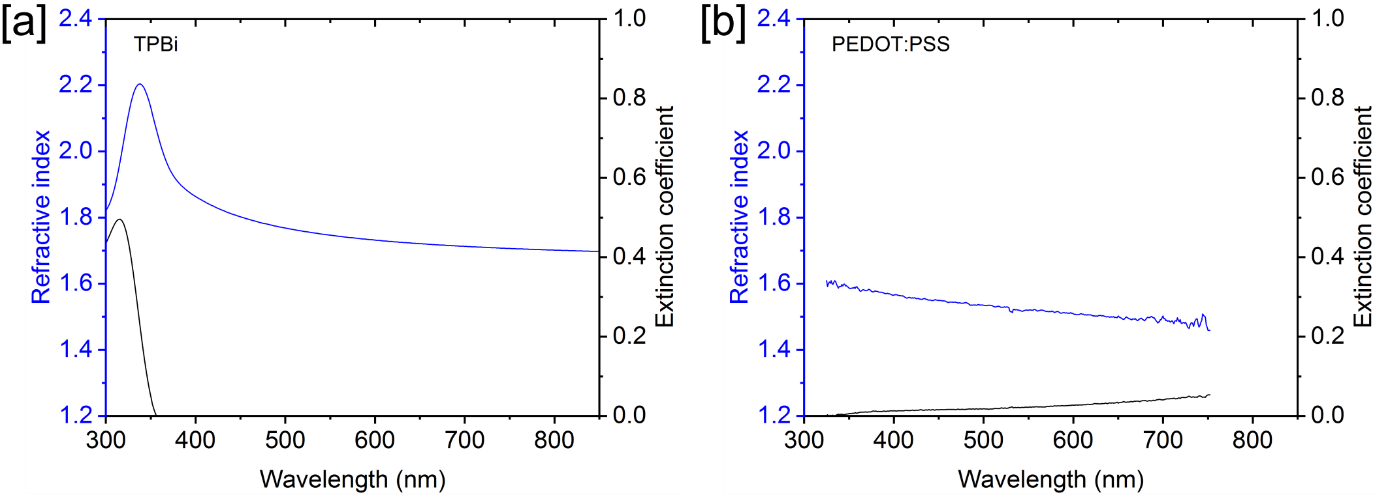


Figure S5. The refractive index and extinction coefficient of TPBi (a) and PEDOT:PSS (b) for outcoupling efficiency calculation in OLEDs.

Supplementary references:

[1] J. R. Lakowicz, *Principles of Fluorescence Spectroscopy*, New York, **2006**.

[2] F. B. Dias, T. J. Penfold, A. P. Monkman, *Methods Appl. Fluoresc.* **2017**, *5*.

[3] F. B. Dias, J. Santos, D. R. Graves, P. Data, R. S. Nobuyasu, M. A. Fox, A. S. Batsanov, T. Palmeira, M. N. Berberan-Santos, M. R. Bryce, A. P. Monkman, *Adv. Sci.* **2016**, *3*, 1.

[4] Q. Wei, P. Kleine, Y. Karpov, X. Qiu, H. Komber, K. Sahre, A. Kiriy, R. Lygaitis, S. Lenk, S. Reineke and B. Voit, *Adv. Funct. Mater.*, 2017, **27**, 1–11.
